# Prevalence and correlates of depressive symptoms among matriculated university students in Singapore during Covid-19 pandemic: findings from a repeated cross-sectional analysis

**DOI:** 10.1186/s12889-024-17866-7

**Published:** 2024-02-13

**Authors:** Pearlie Mei En Yeo, Vicky Mengqi Qin, Chin-Siang Ang, Michael Chia, Ringo Moon-Ho Ho, Andy Hau Yan Ho, Josip Car

**Affiliations:** 1 Lee Kong Chian School of Medicine, Nanyang Technological University, Singapore, Singapore; 2 Centre for Population Health Sciences, Lee Kong Chian School of Medicine, Nanyang Technological University, Singapore, Singapore; 3 Primary Care and Family Medicine programme, Lee Kong Chian School of Medicine, Nanyang Technological University, Singapore, Singapore; 4 Physical Education & Sports Science, National Institute of Education, Nanyang Technological University, Singapore, Singapore; 5 Psychology Programme, School of Social Sciences, Nanyang Technological University, Singapore, Singapore; 6 Department of Primary Care and Public Health, School of Public Health, Imperial College London, London, UK

**Keywords:** Depression, Covid-19, University student, Prevalence, Determinant

## Abstract

**Background:**

Depression is a common issue among university students and has been particularly exacerbated during the COVID-19 pandemic. However, limited research has specifically focused on depression among university entrants.

**Objectives:**

This study aimed to determine the prevalence of depression severity and identify associated factors during different phases of the COVID-19 pandemic using health screening questionnaires completed by matriculated university students in Singapore.

**Methods:**

A repeated cross-sectional study was conducted at a public university in Singapore. Data from health screening questionnaires administered in 2020 and 2021, involving 15,630 newly enrolled university students, were analyzed. The questionnaires covered students’ sociodemographic information, physical health status, own and family medical history, lifestyle behaviours, and the Patient Health Questionnaire (PHQ-9). The PHQ-9 was used to measure the severity of depressive symptoms, categorizing into moderate to severe depressive symptoms (MSDS), mild depressive symptoms (MDS), or no depressive symptom (NDS). Multinomial logistic regression was used to assess the sociodemographic, physical and behavioural correlates of depression.

**Results:**

The prevalence of MSDS was 1% in both 2020 and 2021, while the rates for MDS were 1.93% in 2020 and 1.64% in 2021. In the 2020 cohort, male freshmen who reported better health had a lower likelihood of experiencing depression. Conversely, students of Malay ethnicity, those majoring in Engineering, those with multiple chronic diseases, monthly alcohol consumers, current smokers, and those with a family history of mental disorder had a higher likelihood of experiencing depression. Moreover, students who lived on-campus in the 2021 cohort were less likely to experience depression than those living off-campus. However, the associations between academic majors, alcohol consumption, and smoking with depression were not significant in the 2021 cohort.

**Conclusions:**

This study reported a low prevalence of both MSDS and MDS among university entrants in Singapore. The study further identified three categories of factors associated with depression: sociodemographic, physical, and behavioural. This study suggests policy interventions to enhance targeted social support that address each student group’s specific requirements and susceptibilities. A more extensive and comprehensive study is warranted to assess the changes in student mental health status post-COVID-19 pandemic.

## Background

Entering university is an exciting journey of exploration and personal growth for young adults. However, this experience can be hindered by depression, a common mental health problem that causes persistent low mood and diminished pleasure. According to the World Health Organization (WHO)’s Mental Health Survey, the global 12-month prevalence of major depressive disorders among university students aged between 18 and 22 years varied from 4.5 to 7.7% [1]. Depression can impair young adults’ functioning in various domains, such as substance use, gambling behaviour, sleep quality, and academic achievement, relative to their non-depressed counterparts [2–4]. 

The diversity and complexity of depressive experiences and outcomes depend on various factors. Some of these factors are sociodemographic, such as age, gender, ethnicity, study level, and residency status [5, 6]. Sociodemographic characteristics may affect the level of exposure, vulnerability, and resilience to depressive triggers and stressors. Behavioural factors, such as alcohol and tobacco use, [2, 5] may indicate maladaptive coping strategies, impaired emotion regulation, or increased rumination, which are all associated with higher depression risk. Additionally, physical factors such as multimorbidity, family history of depression, and obesity could also contribute to depression [7–9]. These physical factors may influence the neurobiological, psychological, and social mechanisms that underlie depression, either directly or indirectly. Therefore, it is important to comprehensively examine the sociodemographic, physical and behavioural factors when assessing depression among young adults.

The outbreak of coronavirus disease (COVID-19) since 2019 has been associated with a rise in depression among various populations, particularly among young adults. Singapore, a highly urbanized country, has one of the highest rates of depression in the Asia-Pacific region [10–12]. The WHO report, “Mental Health and Covid: Early Evidence of the Pandemic’s Impact,” indicates that the global prevalence of depression increased by 27.6% from 2019 to 2020, with the highest burden among those aged 20–24 years [13]. To contain the spread of COVID-19 since the first case reported in January 2020, the Singapore government implemented a nationwide lockdown measure known as “circuit breaker” between 3rd April and 2nd June 2020 [14]. After a gradual reopening in the second half of 2020, a second wave of infections due to Delta variant led to longer and more repeated changes of preventive measures from 8th May until zero-Covid strategy been phased out in October 2021 [15, 16]. These measures have not only affected the educational activities of university students, but also heightened their worries about career prospects, infection risk, and social and interpersonal relationships. All these factors could have a profound impact on their mental health.

Depression is a treatable and preventable mental disorder that can have serious consequences for health and well-being if left undiagnosed and untreated [17]. Early detection and intervention can prevent the development of chronic depression and reduce the risk of other comorbid conditions. Previous epidemiological studies have not sufficiently documented the magnitude and determinants of depression among young adults during the pandemic [18, 19]. It is essential to identify the contributors of the occurrence of depression among young adults and to provide them with adequate mental health services and support. One possible way to achieve this is to implement a matriculation screening program that uses self-administered surveys to assess the mental health status and needs of new university entrants. To monitor and promote student health and well-being, some countries have implemented mandatory health screening for tertiary education applicants [20, 21]. Health screening allows for the collection of baseline health data and the evaluation of health outcomes during the academic years. In the current study, using data from the health screening program for two batches of new university entrants enrolled in different phases of the COVID-19 pandemic in Singapore, we (1) measure the prevalence of depressive symptoms among two batches of new university entrants, (2) assess the correlates of different levels of severity of depressive symptoms among university entrants.

We contribute to the literature of depression research in several ways. First, we add to the evidence of the prevalence of depressive symptoms and the change in the prevalence among university entrants during different phases of the COVID-19 pandemic. Second, we present data on a large sample of university entrants from a health screening program that contains comprehensive measurements of sociodemographic, physical and behavioural factors. Third, the health screening program was repeatedly conducted during the pandemic in 2020 to 2021, where the severity of both the pandemic and corresponding preventive measures was different. This facilitates the understanding of how the correlates of depression differ across different phases of the pandemic.

## Methods

### Data and participants

This study was carried out at a national university in Singapore from 2020 to 2021. We aimed to recruit the entire cohort of new students who matriculated from May to early August in both years. As part of the mandatory health screening process, students who visited the on-campus health clinic were asked to complete a digital health survey for this study. A total of 15,630 new students consented to participate in the health survey over two years. 8,575 and 6,984 students completed the survey in 2020 and 2021 respectively. The survey measured students’ self-reported demographics, physical health, personal and family medical history, lifestyle behaviours, and the Patient Health Questionnaire (PHQ-9). All materials and the informed consent form used in this study were reviewed and approved by the medical officers of the health clinic.

### Measurement of depression

To measure the presence and severity of depressive symptoms, we administered the PHQ-9, a nine-item scale based on the nine criteria from the Statistical Manual of Mental Disorders (DSM-IV) [22]. Previous studies have shown that the scale has good validity (sensitivity of 91.7% and specificity of 72.2%) and reliability (Cronbach’s alpha 0.87) in a primary care setting in Singapore [23]. Participants indicated how often they experienced each depressive symptom in the past two weeks on a scale of “not at all”, “several days”, “more than half the days” and “nearly every day”, with scores ranging from 0 to 3. Kroenke et al. proposed two different strategies to interpret the PHQ-9 scores based on synthesized evidence: (1) a cut-off value of 10 for the sum of item scores (ranged from 0 to 27) indicates whether the person has a positive screen for depression, or (2) a diagnostic algorithm with a score of 2 or more in at least five items, and one of the items must include item 1 (anhedonia) or 2 (depressed mood) [24]. In this study, we opted for the latter method, due to the inconsistent evidence about the validity of the cut-off value [25, 26]. A meta-analysis of 18 validation studies did not find significant differences in the pooled sensitivity and specificity of the different PHQ-9 cut-off points (i.e., 8 to 11) [27]. Previous studies adopting different cut-off points were dependent on the population they assessed [28, 29]. Although the cut-off value of 10 and above on the PHQ-9 has been widely used, it was found to overestimate the prevalence of depressive symptoms substantially. Compared to the cut-off values, a PHQ-9 algorithm is relatively consistent across prior studies to classify participants into different risk profiles of depression [30]. Based on this algorithm, a categorical variable was generated for the severity of depressive symptoms, which included moderate to severe depressive symptoms (MSDS), mild depressive symptoms(MDS), and no depressive symptom(NDS) [24]. MSDS was defined as having five or more symptoms (at least one of which must be anhedonia or depressed mood) for more than half days (except for suicidal ideation, which was counted as a symptom if present for several days or more). MDS was defined as having two to four symptoms (must include either anhedonia or depressed mood) for more than half days (except for suicidal ideation, which was counted as a symptom if present for several days or more). NDS was defined as not meeting the criteria for MSDS and MDS.

### Sociodemographic, physical and behavioural factors

To examine the correlates of depression among university entrants, we designed the survey comprising of questions on sociodemographic, physical and behavioural factors that may influence mental health outcomes. The *sociodemographic factors* included gender (male, female), ethnicity (Chinese, Malay, Indian, others), residency status (citizen, permanent resident, foreigner), accommodation type (off-campus, on-campus), matriculation year (2020, 2021), study level (undergraduate, postgraduate), and major (social science, medicine and science, engineering). Students from arts, business, education, humanities, communication, political science, and other social sciences programs were classified as “social science” majors. Subjects other than social science, medicine and engineering were classified as “science” major.

The *physical factors* included self-rated health (four-point Likert scale from worst to best), physical multimorbidity (none, one, more than one), obesity (normal, underweight, overweight, obese), and family history of mental disorder (yes, no). Self-rated health was measured by a single question “How would you rate your current health (over the last 2 weeks)?” that that asked students to rate their current health over the last two weeks on a scale ranging from 0 to 100, where 0 indicated the “worst possible health” and 100 indicated the “best possible health”. The responses were divided into quartiles (first quartile is the worst and fourth quartile is the best). Physical multimorbidity was assessed by asking students to report whether they had ever been diagnosed by a doctor with any of the following 13 diseases: brain or nervous system disorders, eye problems, ear-nose-throat problems, breathing or respiratory disorders, heart or cardiovascular disorders, gastrointestinal disorders, genital-urinary problems, endocrine disorders, muscle or skeletal problems, skin problem, blood disorders, chronic infections, and cancer. The sum of the diseases was categorized as none, one, or more than one. We measured obesity by using body mass index (BMI) which was classified as normal, underweight, overweight, or obese based on the World Health Organization criteria for Asians [31]. Family history of mental disorders included dementia, depression, bipolar disorder, anxiety, schizophrenia, and alcohol or substance abuse. Lastly, the *behavioural factors* included smoking status (non-smoker, ever-smoker, current smoker) and alcohol consumption (never, once or less per month, twice or more per month).

### Sample size and statistical analysis

We excluded participants with missing data on depression, sociodemographic, physical, and behavioural factors from the analysis. The final sample for analysis consisted of 15,177 students, including 8,405 from the 2020 intake and 6,772 from the 2021 intake. Descriptive analyses were conducted to illustrate the prevalence of depression, followed by the sociodemographic, physical and behavioural characteristics by depression status. We selected variables adjusted for in the regression analysis based on the significant differences of the chi-squared tests (or Fisher’s exact test if the sample size in the subgroup was less than 5) between the sociodemographic, physical and behavioural variables and depression outcomes. We first adopted ordinal logistic regression to explore the factors associated with depression. A post-estimation test including Brant test, score test, likelihood ratio and Wald statistics suggested the parallel lines assumption is unlikely to hold for the relationship between different categories of the depression outcome and the predicting variables at a 5% significance level. Hence, we used multinomial logistic regression models that does not require parallel lines assumption​. We considered a *p*-value of 0.05 as statistically significant. All statistical analyses were conducted using Stata 16.0.

## Results

### Characteristics of the student cohorts and their mental health prevalence

During the COVID-19 pandemic, the prevalence of MSDS was 1% in both 2020 and 2021, and 1.93% and 1.64% for MDS in 2020 and 2021, respectively. There was no significant difference in the prevalence of depression in 2020 and 2021 (Table 1).

**Table 1:** Prevalence of depression severity in 2020 and 2021

	2020		2021		Risk ratio ^a^	*P* value ^a^
	n	%	n	%		
NDS	8,159	97.07	6,593	97.36	Ref	-
MDS	162	1.93	111	1.64	0.850	0.216
MSDS	84	1.00	68	1.00	0.980	0.909

Note: ^a^ Pooled multinomial logistic-regression analyses with intake (0 = year 2020, 1 = year 2021) treated as a primary predictor of depression after controlling for other social, physical and lifestyle factors. NDS = No depressive symptom; MDS = Mild depressive symptoms; MSDS = moderate to severe depressive symptoms

The sociodemographic, physical and behavioural characteristics of the students and the chi-squared test results are shown in Tables 2 and 3. The mean age was 21.9 years (SD = 4.20) and 22.2 years (SD = 4.85) for the 2020 and 2021 cohorts, respectively. In the 2020 cohort, gender was evenly distributed with 50.1% being female, while the majority of the students were Chinese (83.9%), local citizens (60.9%), undergraduate (67.9%), majoring in social science subjects (45.0%), staying off-campus (67.5%), having no chronic disease (72.2%), having no family history of mental disorders (96.4%), self-reporting good (38.3%) health status, normal BMI (51.9%), non-drinker (52.6%) and non-smoker (95.2%). The notable difference in the characteristic distribution (*P* < 0.01) compared with NDS group was found in: more female (60.5% in MDS and 71.4% in MSDS), local citizens (81.5% in MDS and 88.1% in MSDS), undergraduate level students (88.9% in MDS and 96.4% in MSDS), multimorbidity of more than one chronic disease (19% in MDS and 19.1% in MSDS), worst health status (51.9% in MDS and 57.1% in MSDS), alcohol drinker (60.5% in MDS and 59.5% in MSDS) and current smoker (3.1% in MDS and 8.3% in MSDS), and with family history of mental disorder (8.6% in MDS and 8.3% in MSDS). The characteristic distribution was similar for the 2021 cohort compared with the 2020 cohort. The exception was found in the distribution of ethnicity, major and accommodation where the difference became statistically significant, whilst the difference in alcohol consumption and smoking status became insignificant.

**Table 2:** Sociodemographic characteristics of student participants

	2020 (*N* = 8,405)									2021 (*N* = 6,772)								
	Total		NDS		MDS		MSDS		*P* value	Total		NDS		MDS		MSDS		*P* value
	n	%	n	%	n	%	n	%		n	%	n	%	n	%	n	%	
**Age**, mean (SD)^**+**^	21.9	4.20	21.9	4.23	20.8	3.16	20.3	1.91	0.067^§^	22.2	4.85	22.2	4.9	21.4	4.2	20.1	1.99	0.164^§^
**Gender**																		
Female	4,208	50.10	4,050	49.60	98	60.50	60	71.40	**< 0.001**	3,123	46.10	3,005	45.60	68	61.30	50	73.50	**< 0.001**
Male	4,197	49.90	4,109	50.40	64	39.50	24	28.60		3,649	53.90	3,588	54.40	43	38.70	18	26.50	
**Ethnicity**																		
Chinese	7,055	83.90	6,859	84.10	134	82.70	62	73.80	0.056	5,797	85.60	5,662	85.90	84	75.70	51	75.00	**< 0.001**
Malay	300	3.60	289	3.50	4	2.50	7	8.30		315	4.70	296	4.50	11	9.90	8	11.80	
Indian	597	7.10	578	7.10	13	8.00	6	7.10		382	5.60	374	5.70	6	5.40	2	2.90	
other	453	5.40	433	5.30	11	6.80	9	10.70		278	4.10	261	4.00	10	9.00	7	10.30	
**Residency status**																		
Citizen	5,115	60.90	4,909	60.20	132	81.50	74	88.10	**< 0.001**	5,174	76.40	5,022	76.20	92	82.90	60	88.20	**0.01**
Permanent Resident	456	5.40	446	5.50	5	3.10	5	6.00		396	5.80	383	5.80	9	8.10	4	5.90	
Foreigner	2,834	33.70	2,804	34.40	25	15.40	5	6.00		1,202	17.70	1,188	18.00	10	9.00	4	5.90	
**Study level**																		
Undergraduate	5,711	67.90	5,486	67.20	144	88.90	81	96.40	**< 0.001**	5,000	73.80	4,842	73.40	94	84.70	64	94.10	**< 0.001**
Postgraduate	2,694	32.10	2,673	32.80	18	11.10	3	3.60		1,772	26.20	1,751	26.60	17	15.30	4	5.90	
**Major**																		
Social Science	3,780	45.00	3,665	44.90	69	42.60	46	54.80	0.133	3,187	47.10	3,091	46.90	56	50.50	40	58.80	**0.027**
Medicine & Science	1,254	14.90	1,218	14.90	21	13.00	15	17.90		1,139	16.80	1,105	16.80	18	16.20	16	23.50	
Engineering	3,371	40.10	3,276	40.20	72	44.40	23	27.40		2,446	36.10	2,397	36.40	37	33.30	12	17.60	
**Accommodation**																		
Off-campus	5,674	67.51	5,518	67.63	98	60.49	58	69.05	0.151	5,597	82.65	5,445	82.59	102	91.89	50	73.53	**0.005**
On-campus	2,731	32.49	2,641	32.37	64	39.51	26	30.95		1,175	17.35	1,148	17.41	9	8.11	18	26.47	

Note: ^**+**^ The sample size for calculating the age mean was 8,363 for 2020 and 6,754 for 2021. Due to the collinearity of age and study level, we did not control for age in the later regression analysis, therefore we did not remove samples that have only missing on age data. ^§^*P* value was generated based on one-way ANOVA test. *P* values that are significant were bolded. NDS = No depressive symptom; MDS = Mild depressive symptoms; MSDS = moderate to severe depressive symptoms

**Table 3:** Physical and behavioural characteristics of student participants

	2020 (*n* = 8,405)									2021 (*n* = 6,772)								
	Total		NDS		MDS		MSDS		*P* value	Total		NDS		MDS		MSDS		*P* value
	n	%	n	%	n	%	n	%		n	%	n	%	n	%	n	%	
**Multimorbidity**																		
None	6,068	72.20	5,939	72.80	89	54.90	40	47.60	**< 0.001**	4,714	69.60	4,621	70.10	65	58.60	28	41.20	**< 0.001**
One chronic disease	1,737	20.70	1,667	20.40	42	25.90	28	33.30		1,500	22.20	1,444	21.90	31	27.90	25	36.80	
More than one chronic disease	600	7.10	553	6.80	31	19.10	16	19.00		558	8.20	528	8.00	15	13.50	15	22.10	
**Health status**																		
1 (worst)	2,140	25.50	2,008	24.60	84	51.90	48	57.10	**< 0.001**	1,771	26.20	1,678	25.50	51	45.90	42	61.80	**< 0.001**
2	3,040	36.20	2,969	36.40	46	28.40	25	29.80		2,561	37.80	2,502	37.90	43	38.70	16	23.50	
3	1,146	13.60	1,128	13.80	16	9.90	2	2.40		786	11.60	777	11.80	6	5.40	3	4.40	
4 (best)	2,079	24.70	2,054	25.20	16	9.90	9	10.70		1,654	24.40	1,636	24.80	11	9.90	7	10.30	
**Obesity**																		
Normal	4,360	51.90	4,237	51.90	83	51.20	40	47.60	0.913	3,344	49.40	3,252	49.30	54	48.60	38	55.90	0.77
Underweight	1,242	14.80	1,204	14.80	23	14.20	15	17.90		891	13.20	866	13.10	18	16.20	7	10.30	
Overweight	2,064	24.60	2,000	24.50	44	27.20	20	23.80		1,831	27.00	1,790	27.20	26	23.40	15	22.10	
Obese	739	8.80	718	8.80	12	7.40	9	10.70		706	10.40	685	10.40	13	11.70	8	11.80	
**Alcohol consumption**																		
Never	4,422	52.60	4,324	53.00	64	39.50	34	40.50	**0.001**	3,368	49.70	3,287	49.90	51	45.90	30	44.10	0.649
Once or less monthly	3,329	39.60	3,206	39.30	84	51.90	39	46.40		2,949	43.50	2,866	43.50	52	46.80	31	45.60	
Twice or more monthly	654	7.80	629	7.70	14	8.60	11	13.10		455	6.70	440	6.70	8	7.20	7	10.30	
**Smoking**																		
Non-smoker	8,003	95.20	7,775	95.30	154	95.10	74	88.10	**0.018**	6,424	94.90	6,256	94.90	106	95.50	62	91.20	0.540
Ever smoker	204	2.40	198	2.40	3	1.90	3	3.60		204	3.00	197	3.00	3	2.70	4	5.90	
Current smoker	198	2.40	186	2.30	5	3.10	7	8.30		144	2.10	140	2.10	2	1.80	2	2.90	
**Family history of mental disorder**																		
No	8,101	96.40	7,876	96.50	148	91.40	77	91.70	**< 0.001**	6,473	95.60	6,323	95.90	95	85.60	55	80.90	**< 0.001**
Yes	304	3.60	283	3.50	14	8.60	7	8.30		299	4.40	270	4.10	16	14.40	13	19.10	

Note: Independence test for health status groups and smoking groups were conducted using Fisher’s exact test. *P* values that are significant were bolded. NDS = No depressive symptom; MDS = Mild depressive symptoms; MSDS = moderate to severe depressive symptoms

### Association of social, behavioural, and physical factors and depression

Table 4 presents the associations between sociodemographic, physical and behavioural correlates and depression. In the 2020 cohort, being male (RR = 0.52, 95% CI = 0.367–0.746 for MDS; RR = 0.33, 95% CI = 0.193–0.570 for MSDS), enrolled in an undergraduate program (RR = 0.30, 95% CI = 0.181–0.496 for MDS; RR = 0.081, 95% CI = 0.025–0.260 for MSDS) and self-reported better health status (RR = 0.22, 95% CI = 0.129–0.383 for MDS; RR = 0.23, 95% CI = 0.113–0.481 for MSDS) were less likely to have depression compared to their counterparts.

**Table 4:** Sociodemographic, physical and behavioural correlates of depression from multinomial logistic regression in 2020 and 2021

	2020 (*n* = 8,405)		2021 (*n* = 6,772)	
Ref: NDS	MDS	MSDS	MDS	MSDS
	Risk ratio (95% Confidence interval)			
**Sociodemographic**				
**Gender**				
Female	ref	ref	ref	ref
Male	0.523***	0.332***	0.493***	0.324***
	(0.367–0.746)	(0.193–0.570)	(0.318–0.763)	(0.176–0.596)
**Ethnicity**				
Chinese	ref	ref	ref	ref
Malay	0.872	2.935**	2.794***	3.433***
	(0.311–2.441)	(1.250–6.888)	(1.401–5.572)	(1.487–7.927)
Indian	1.417	1.643	1.337	0.799
	(0.783–2.566)	(0.690–3.912)	(0.572–3.124)	(0.189–3.373)
Other	1.402	2.770***	2.557***	2.065
	(0.744–2.644)	(1.334–5.751)	(1.282–5.099)	(0.850–5.016)
**Study level**				
Undergraduate	ref	ref	ref	ref
Postgraduate	0.300***	0.081***	0.487***	0.161***
	(0.181–0.496)	(0.025–0.260)	(0.284–0.836)	(0.057–0.457)
**Major**				
Social sci	ref	ref	ref	ref
Medicine & science	0.801	0.842	0.909	1.14
	(0.483–1.328)	(0.460–1.541)	(0.523–1.579)	(0.621–2.093)
Engineering	1.420*	0.657	1.054	0.56
	(0.981–2.056)	(0.379–1.140)	(0.658–1.689)	(0.276–1.137)
**Accommodation**				
Off campus	ref	ref	ref	ref
On campus	1.249	0.826	0.354***	1.242
	(0.902–1.730)	(0.511–1.333)	(0.177–0.710)	(0.698–2.208)
**Physical**				
**Multimorbidity**				
None	ref	ref	ref	ref
One chronic disease	1.357	1.942***	1.338	2.378***
	(0.930–1.982)	(1.181–3.195)	(0.861–2.078)	(1.361–4.154)
More than one chronic disease	2.556***	2.706***	1.356	2.732***
	(1.661–3.933)	(1.473–4.973)	(0.754–2.440)	(1.398–5.340)
**Health status**				
1 (worst)	ref	ref	ref	ref
2	0.405***	0.402***	0.581**	0.276***
	(0.280–0.585)	(0.245–0.659)	(0.383–0.881)	(0.153–0.498)
3	0.400***	0.092***	0.269***	0.163***
	(0.231–0.690)	(0.022–0.380)	(0.114–0.632)	(0.050–0.533)
4 (best)	0.222***	0.233***	0.234***	0.218***
	(0.129–0.383)	(0.113–0.481)	(0.121–0.454)	(0.096–0.494)
**Family history of mental disorder**				
No	ref	ref	ref	ref
Yes	1.704*	1.337	2.927***	2.680***
	(0.957–3.033)	(0.596–3.000)	(1.651–5.188)	(1.374–5.228)
**Behavioural**				
**Alcohol consumption**				
Never	ref	ref	ref	ref
Once or less monthly	1.691***	1.588*	1.37	1.258
	(1.199–2.385)	(0.969–2.602)	(0.899–2.086)	(0.726–2.182)
Twice or more monthly	1.354	1.782	1.43	1.591
	(0.732–2.505)	(0.831–3.819)	(0.642–3.187)	(0.603–4.193)
**Smoking**				
Non-smoker	ref	ref	ref	ref
Ever smoker	0.712	1.586	0.79	2.368
	(0.218–2.324)	(0.461–5.461)	(0.237–2.634)	(0.733–7.653)
Current smoker	1.383	4.655***	0.732	1.41
	(0.536–3.571)	(1.887–11.481)	(0.170–3.157)	(0.292–6.799)

Note: *** *p* < 0.01, ** *p* < 0.05. NDS = No depressive symptom; MDS = Mild depressive symptoms; MSDS = moderate to severe depressive symptoms

On the other hand, students being Malay ethnic (RR = 0.87, 95% CI = 0.311–2.441 for MDS; RR = 2.93, 95% CI = 1.250–6.888 for MSDS), majoring in Engineering subjects (RR = 1.42, 95% CI = 0.981–2.056 for MDS; RR = 0.66, 95% CI = 0.379–1.140 for MSDS), having more than one chronic disease (RR = 2.56, 95% CI = 1.661–3.933 for MDS; RR = 2.71, 95% CI = 1.472–4.973 for MSDS), consuming alcohol every month (RR = 1.69, 95% CI = 1.199–2.385 for MDS; RR = 1.59, 95% CI = 0.969–2.602 for MSDS), currently smoking (RR = 1.38, 95% CI = 0.536–3.571 for MDS; RR = 4.66, 95% CI = 1.887–11.481 for MSDS), and with a family history of mental disorder (RR = 1.70, 95% CI = 0.957–3.033 for MDS; RR = 1.34, 95% CI = 0.596–3.00 for MSDS) were more likely to have depression, holding other variables constant.

The direction of the association between the abovementioned factors (i.e., gender, study level, multimorbidity, self-reported health status) and depression remained in the 2021 cohort. It is worth noting that the scale of the likelihood of having depression was larger for students who were Malay ethnic, with multimorbidity of chronic diseases, had family history of mental disorder, and were undergraduate level students in the 2021 cohort compared to the 2020 cohort. In addition, students in the 2021 cohort who stayed on-campus were less likely to have depression compared to those who stayed off-campus (RR = 0.35, 95% CI = 0.177–0.710 for MDS; RR = 1.24, 95% CI = 0.698–2.208 for MSDS). The association between type of majors, alcohol consumption, and smoking became insignificant in the 2021 cohort.

## Discussion

This study investigated the prevalence of depression and correlates of depression among university entrants in Singapore during the COVID-19 pandemic, using data from two repeated cross-sectional surveys conducted in 2020 and 2021. The results showed that the prevalence of MSDS was 1.00% in both cohorts, and the prevalence of MDS was slightly lower in 2021 (1.64%) than in 2020 (1.93%). These rates were remarkably lower than those reported in the previous studies on university students during the pandemic, which ranged from 29.4 to 40.9% [32–34]. The possible explanation for this discrepancy could include the differences in the study population (i.e., university entrants versus existing students), the scoring strategy, and the cultural and contextual factors. Our study population were newly enrolled students to university who might not have experienced tertiary education environment as existing students [21]. Moreover, the PHQ-9 algorithm applied in this study was more rigorous than the cut-off scores employed for PHQ-9 or other instruments for measuring depression in other studies, which could have led to a deflated prevalence of depression [35]. Culturally, the stigma and fear associated with mental health issues in Singapore might have deterred some students from disclosing their true condition during matriculation health screening [36]. 

We observed that being female, Malay ethnic and undergraduate level were significantly associated with a higher risk of depression in both 2020 and 2021 cohorts. The finding of gender difference is consistent with previous studies, which could be attributed to biological factors such as brain structure and hormonal fluctuations, [37, 38] and psychosocial factors such as gender roles and social expectations [36]. The vulnerability of ethnic minority students in developing MSDS may reflect the phenomenon of “Chinese privilege” in Singapore, which refers to the perceived advantages that the ethnic majority enjoy over the ethnic minorities, such as better access to resources, opportunities, and recognition [39]. Additionally, a higher risk of developing both MDS and MSDS among undergraduate students than postgraduate students could be due to the lower stress coping skills and higher academic pressure experienced by undergraduate students [40]. 

Students enrolling in engineering major had a higher risk of developing MDS than students from other majors. The relationship between depression and academic major remains inconclusive and we argue that the severity of depression could associate with the characteristics and culture of the university and discipline [41, 42]. Our study sample matriculated to a university where engineering is a prestigious and competitive major. Students enrolled to this major are likely to experience higher level of stress thereafter higher risk of depression. We found that 2021 intake students who lived on campus had a lower risk of MDS than those who lived off campus, whereas no such difference was detected in the 2020 cohort. This could be due to the more readily available services and supports for students’ psychological wellbeing on-campus in the second year of pandemic, which could buffer the negative effects of stress and isolation [43]. 

In terms of physical factors, the results revealed similar findings between the two cohorts for multimorbidity, self-rated health status, and family history of mental disorders. Specifically, the occurrence of multimorbidity and family history of mental disorders were risk factors, while better self-rated health status was a protective factor for depression. Previous research has suggested that anatomical variations may mediate the effect of family history on depression risk by influencing brain structure and function [8, 44]. Furthermore, chronic diseases may contribute to depression due to the emotional distress and functional impairment [45]. Likewise, those with worse self-reported health status who indicate a poorer subjective perception of their social, physical, and psychological health, tend to have a more pessimistic view of their experiences, future, and self, resulting in higher susceptibility to depression [9]. 

Of the behavioural correlates, it was observed that smoking and alcohol consumption were significantly associated with higher depression prevalence in 2020, but not in 2021. Stricter lockdown policies in the first year of the pandemic could partly explain the increased stress levels and coping behaviours among residents, especially smokers who may perceive smoking as a way to relieve tension. Previous studies supported that substance use and mental health problems exacerbated after public threats such as terrorist attacks, natural disasters, and COVID-19 outbreaks [46]. With better prepared response to disease outbreaks in 2021, Singapore adopted a more flexible, less stringent but more preventive measures such as wearing masks, maintaining social distance, and contact tracing [16]. This may have reduced the psychological impact of the pandemic and moderated the relationship between substance use and depression [47–49]. 

More broadly, this study provides further evidence that the prevalence of depression among university entrants during the pandemic is influenced by different risk factors that vary in their magnitude and persistence over time. We found that the relationship between sociodemographic factors (ethnicity, study level and accommodation) and higher depression prevalence were stronger in the 2021 cohort than the 2020 cohort. This indicates that the pandemic has exacerbated the existing inequities and vulnerabilities of certain subpopulations, [6] including ethnic minorities, undergraduates and students staying off-campus. The physical factors that were associated with higher depression prevalence in both cohorts had a higher relative risk in the 2021 cohort, implying that the ongoing pandemic has worsened the negative impact of the pre-existing health conditions on mental health outcomes [18]. The behavioural factors that were associated with higher depression prevalence in the 2020 cohort had a weaker relative risk in the 2021 cohort, which could be attributed to a coping and adaptive mechanism in response to the acute stress following the outbreak of the pandemic [2, 5]. Despite the limitations of using two cross-sectional samples, this study adds to the existing literature by demonstrating that sociodemographic and physical risk factors have a more lasting and accumulated influence on depression among university entrants during the pandemic, while behavioural risk factors have a more immediate and transient influence at the onset of the pandemic.

### Strengths and implication

Our findings provide a snapshot of the depression prevalence among the university entrants during different phases of the pandemic, and its relationship with sociodemographic, physical and behavioural factors. Our study also fills an evidence gap on mental health research on university entrants who have not yet started their tertiary education. We observed that university entrants with different sociodemographic backgrounds and with different physical risk factors exhibited varying degrees of depression, and some of the factors showed lasting and accumulated influence in the second year of the pandemic. This study was not able to conclude the impact of the pandemic on depression comparing pre-pandemic period due to the lack of pre-pandemic data. However, compared to the first year of the pandemic, when the lockdown policy during which the students matriculated, had shorter and expected period of implementation, the longer and more repeated changes of preventive measures in the second year of the pandemic may have more negative impact on those who were already at the vulnerable groups [16]. 

Interventions on students’ mental well-being should target primordial prevention, at the time of matriculation, that account for students’ diverse sociodemographic and physical characteristics, while planning for future similar disruptions. Another crucial strategy is to monitor and timely intervention to reduce substance use including smoking and alcohol consumption of students who experienced depression. These students may require additional support and guidance to manage stress and anxiety in difficult times. Measurement tool wise, the use of a more conservative PHQ-9 algorithm for depression prevalence estimation should also be considered to avoid overestimation, of which false-positive screening results are not only worsen prognosis but also cause emotional distress due to the fear of stigma and discrimination.

### Limitations

This study has some limitations that warrant caution in interpreting the findings. First, we used the PHQ-9, a self-report instrument that assesses the presence and severity of depressive symptoms but does not provide a clinical diagnosis of depression [23]. Although semi-structured or structured interviews are the gold standard for diagnosing depression, they are impractical for screening large cohorts of university entrants. Second, we employed a cross-sectional study design that captured the data at one point in time during the pandemic, which precluded us from comparing the depression rates and risk factors before and after the pandemic. A longitudinal design following the same cohort would have been ideal to examine the temporal changes in depression outcomes, but it was not feasible given the logistical challenges of conducting such a study with this population during a global health crisis. Therefore, we opted for a cross-sectional design that enabled us to collect data from a sizable and diverse sample of university entrants to estimate the prevalence of depression. However, this also means that our analysis can only infer associations and not causality. Third, the sample was drawn from a single university in Singapore and comprised of students of similar ages. This may affect the generalisability of the findings to different age groups and other countries where preventive measures of different intensities or duration were adopted. Future studies can consider recruiting participants from multiple universities and different age groups to increase the representativeness of the sample. Fourth, the health screening in this study only assessed depression, which is one of the common mental health problems among university students. Other prevalent mental health issues, such as anxiety disorders and substance-use disorders [23], were not included in the survey. Lastly, the survey did not measure pandemic-related variables and stressors, as they were beyond the scope of the matriculation health screening. Therefore, we cannot determine whether the differences observed in this study were more pronounced during or before the pandemic.

## Conclusions

This study reported a low prevalence of both MSDS and MDS among matriculated students at a university in Singapore. The study further identified three categories of factors associated with depression: sociodemographic (gender, ethnicity, study level, academic major, and residency status), physical (multimorbidity, self-reported health status, and family history of mental disorder), and behavioural (smoking and alcohol consumption). This study suggests policy interventions to enhance targeted social support that address each student group’s specific requirements and susceptibilities. A more extensive and comprehensive study is warranted to assess the changes in student mental health status after the recovery from the COVID-19 pandemic.

## Data Availability

The datasets generated and/or analysed during the current study are not publicly available. Data are however available from the authors upon reasonable request.

## Abbreviations

BMI: Body Mass Index
DSM-V: Diagnostic and Statistical Manual of Mental Disorders, Fifth Edition
MSDS: Moderate to severe depressive symptoms
NDS: No depressive symptom
PHQ-9: Patient Health Questionnaire
RR: Risk ratio
SRHS: Self-Reported Health Status
MDS: Mild depressive symptoms
